# One year analysis of Prospective Memory Clinics Registry in Qatar: A Critical Tool for Dementia Research and Policy Planning

**DOI:** 10.3126/nje.v14i2.69364

**Published:** 2024-09-02

**Authors:** Hanadi Al Hamad, Navas Nadukkandiyil, Mani Chandran, Pravija Talapan Manikoth, Brijesh Sathian

**Affiliations:** 1 Geriatrics and long term care department, Rumailah Hospital, Hamad Medical Corporation, Doha, Qatar

**Keywords:** Memory Clinic, Dementia, Registry, Research, Qatar

## Abstract

**Background:**

The Global Dementia Observatory (GDO) is a monitoring and accountability tool for the Global Action Plan on Public Response to Dementia 2017–25. Evidence from dementia registries may be utilized to better address WHO efforts in member countries, as well as to improve clinical practice and public health policy. The goal of this study was to analyze one-year data from a prospective memory clinic registry.

**Methods:**

This study was a baseline analysis of prospective memory clinics registry data of Qatar from January 1, 2023, through December 31, 2023.

**Results:**

This study investigated the demographic, clinical, and lifestyle characteristics of 464 participants who were enrolled in memory clinics. Mild neurocognitive disorders were the most prevalent diagnoses in both sexes, affecting 61.5% of male patients and 63.7% of female patients. Dementia was slightly more common in men (19.8% vs. 18.9%), although delirium was more common in women (1.9% vs. 0%). In terms of risk factors, the analysis revealed that females were more likely to be obese (36.8% vs. 16.7% in males), while males had higher rates of diabetes (61.1% vs. 51.9% in females), hypertension (69.4% vs. 62.7% in females), and smoking (17.1% vs. 3.8% in females).

**Conclusion:**

The findings of this study highlight the differences in dementia risk factors between genders and races, highlighting the need for customized interventions. Furthermore, the registry is a great resource for policymakers and healthcare professionals, providing evidence-based suggestions to improve dementia care, increase the well-being of patients and caregivers, and maximize resource allocation.

## Introduction

Dementia, a degenerative neurological disorder, is anticipated to double in incidence every 20 years, rising from 46.8 million in 2015 to over 131.5 million by 2050 [1, 2]. Despite rising concerns, little is known about the natural history of various types of dementia, the quality of diagnosis and management, community resource utilization, long-term care demands, and the economic and emotional burden on caregivers. The current information is based on studies with small sample sizes, which makes it difficult to generalize the findings to the population level. Reliable dementia statistics are crucial, particularly in low- and middle-income nations where such data are sparse.

These findings can help to influence legislation, improve planning, and allocate resources to address the growing dementia burden. It is possible to enhance the quality of life of persons with dementia and their families while simultaneously decreasing the economic burden of Alzheimer's disease and other dementias by applying novel techniques of prevention, treatment, care, and policy reform [3,4]. Dementia registries were used to gather high-quality epidemiological data and to evaluate the quality of dementia care.

According to Jefferson et al., clinical registries can lower yearly healthcare costs while providing a ten-fold return on investment [5]. This finding emphasizes the economic and therapeutic benefits of these registries. Dementia registries will play an important role in future therapeutic trials, because this cohort can be recruited. The data from these registries can be utilized to enhance clinical practice and public health policy [6-16]. The goal of this study was to analyze one-year data from a prospective memory clinic registry.

## Methodology

### Study Design and Participants

This was a baseline analysis of prospective memory clinics registry data from January 1, 2023, through December 31, 2023. All patients aged 60 years and above from memory clinics were included in the study. Patients aged < 60 years and those who were not registered in the memory clinic were excluded.

### Data collection methods

All patients attending the memory clinic underwent a registration process, following which a diagnosis and management plan was made by a multidisciplinary team (routine process). Following each clinic day, the research team extracted data from the clinical evaluations using the Qatar Dementia Registry dataset tool. The collected information was entered into Dendrite software. Specialized data entry software for setting up an electronic registry from the Dendrite’s Intellect Clinical Information System Company, based in the UK, was used. All identifiable data were de-identified by using specific research codes. The link connecting the code and identifier was maintained until data collection was completed. As it is a registry, the software provides long-term data for the purpose of the Qatar National Dementia Plan. We plan to use the non-identifiable data from this study for other projects in the future.

### Ethical committee approval

This study was approved by the Medical Research Centre, the Institutional Review Board of Hamad Medical Corporation (IRGC-05-SI-18-341).

### Data Management and Statistical Analyses

Descriptive statistics was used to analyze the data. All statistical analysis were performed using the R-4.3.2 for Windows software.

## Results

### Gender-wise comparison

The gender-based analysis included 464 patients, including 252 men and 212 females. Among them, a larger proportion of women were Qatari (51.4% vs. 37.7% in men), whereas most males were non-Qatari (62.3% vs. 48.6% in females). Notably, education levels differed considerably across genders, with females being more likely to lack formal schooling (30.7% vs. 11.9% in men), and males being more likely to complete university education (27.8% vs. 17% in females) and postgraduate degrees (11.5% vs. 3.8% in females). Additionally, marital status differed significantly between genders, with 91.3% of males being married compared to 65.1% of females. Furthermore, females had a much higher prevalence of widowhood (18.4% vs. 2.8% in males), which is likely due to gender-based life expectancy differences and cultural factors where men often remarry after the loss of a spouse, whereas women may not. Mild neurocognitive disorders were the most common diagnosis in both sexes, accounting for 61.5% of male patients and 63.7% of female patients. While dementia was slightly more prevalent in males (19.8% vs. 18.9% in females), delirium and post-delirium states were more frequently diagnosed in females (1.9% vs. 0% in males). The occurrence of other diagnoses was comparable between sexes, with both groups having 12.3% and 11.8%, respectively.

A family history of dementia was more frequently reported among females (14.2% vs. 9.9% in males), which might indicate a genetic predisposition or greater awareness and reporting of family history among females. Interestingly, despite the slightly higher rate of psychiatric comorbidities among males (9.9% vs. 8.5% in females), females were more likely to be prescribed antidepressants (20.8% vs. 15.5% in males). This could reflect sex differences in the manifestation of depressive symptoms or a higher likelihood of females seeking or being prescribed treatment. Antipsychotic use was relatively low in both the groups (4.8% in males and 4.2% in females).

Polypharmacy, defined as the use of multiple medications, was more prevalent among males (53.2% vs. 44.3% in females), highlighting a potentially greater burden of comorbidities in male patients. Despite these differences, most patients, regardless of sex, received support from family and friends (95.2% of males and 96.7% of females). However, males were slightly more likely to receive support from private nurses (3.6% vs. 1.9% in females), which could reflect differences in family dynamics or the availability of informal care.

In terms of risk factors, the analysis revealed that females were more likely to be obese (36.8% vs. 16.7% in males), while males had higher rates of diabetes (61.1% vs. 51.9% in females), hypertension (69.4% vs. 62.7% in females), and smoking (17.1% vs. 3.8% in females). Physical inactivity was more common in females (22.2% vs. 16.3% in males), potentially contributing to the higher obesity rates observed. Dyslipidaemia was similarly prevalent in both genders (50% in males vs. 46.7% in females). Hearing loss and traumatic brain injury (TBI) were slightly more common in females (13.7% and 2.8% vs. 12.7% and 0.8% in males, respectively), while social isolation was more common in males (1.2% vs. 0.5% in females).

### Ethnicity-wise Comparisons

Ethnicity analysis, encompassing 204 Qatari and 260 non-Qatari patients, revealed distinct patterns in demographic and clinical characteristics. Non-Qatari males comprised a larger proportion of the sample (60.4% vs. 46.6% of Qatari males), whereas Qatari females were more prevalent (53.4% vs. 39.6% of non-Qatari females). Education levels differed significantly between these groups, with a higher proportion of Qataris with no formal education (31.9% vs. 11.5% in non-Qataris). Conversely, non-Qataris were more likely to have tertiary education (29.6% vs. 14.2% in Qataris) and post-graduate degrees (10.8% vs. 4.4% in Qataris), reflecting the higher educational attainment often observed among expatriates in Qatar.

Geographically, non-Qataris were more likely to reside inside Doha (53.5% vs. 40.2%), while a larger proportion of Qataris lived outside the capital (58.8% vs. 43.5%). The marital status was relatively similar between the two groups, with the majority being married (80.4% of Qataris and 78.5% of non-Qataris), although a higher percentage of Qataris were widowed (12.7% vs. 7.7% in non-Qataris).

Clinically, dementia was more prevalent in Qataris (23.5% vs. 16.2% in non-Qataris), while non-Qataris had higher rates of other diagnoses (15.4% vs. 7.8% in Qataris). Mild neurocognitive disorders were similar in both groups (63.2% in the Qataris vs. 61.9% in the non-Qataris group). The percentage of patients still under evaluation was slightly higher among non-Qataris (5.4% vs. 3.9%), possibly indicating differences in access to or completion of diagnostic assessments.

In terms of psychiatric comorbidities, non-Qataris had a higher prevalence (11.2% vs. 6.9%) and were more likely to be prescribed antidepressants (20.4% vs. 14.7%). Polypharmacy was more common in Qataris (54.4% vs. 45% in non-Qataris), potentially reflecting a higher burden of chronic diseases. However, both groups had strong caregiver support, with over 90% receiving help from family and friends. BPSD was similarly prevalent in both the groups (11.8% in Qataris vs. 11.5% in non-Qataris).

Risk factor analysis revealed that Qataris had higher rates of diabetes (65.2% vs. 50.4% in non-Qataris) and dyslipidemia (55.4% vs. 43.1% in non-Qataris), while non-Qataris were more likely to be physically inactive (20% vs. 17.6% in Qataris) and smokers (14.2% vs. 6.9% in Qataris). Depression was more prevalent among non-Qataris (11.9% vs. 5.4%), possibly indicating different stressors or health-seeking behaviors. Interestingly, Qataris had higher rates of hypertension (70.6% vs. 63.1% in non-Qataris) and hearing loss (13.7% vs. 12.7% in non-Qataris), while traumatic brain injury was more common in Qataris (2.9% vs. 0.8% in non-Qataris).

Overall, the results of this study underscore the importance of considering sex and ethnicity in the management of cognitive disorders, as these factors significantly influence the clinical presentation, risk factors, and access to care among patients attending memory clinics.

## Discussion

### Dementia Registries

The current study provides a comprehensive comparison of memory clinic registry clinical features and risk variables by sex and ethnicity, revealing significant disparities that must be addressed in both clinical practice and public health initiatives. Our registry was established in 2021 using evidence-based epidemiological and clinical data-collection tools. In comparison with our registry, another eight registries gathered epidemiological data on dementia, two with nationwide coverage (Argentina and France) and six that covered a specific region of the country (Italy, Spain, and four registries in the United States) [9–16]. The first epidemiological dementia registries were created in the United States in 1988 and provided state-wide coverage (New York State and South Carolina), with further registers established in 2005. More than half of the dementia registries worldwide intend to perform or facilitate research, such as clinical trials for therapies in the pre-dementia phase of Alzheimer's disease and volunteer recruitment for dementia studies. Other registries gather epidemiological dementia or quality-of-care data. Dementia registries, because of their vast range of applications and objectives, make it easier to diagnose, monitor, and care for persons with dementia, as well as provide caregiver support throughout the disease. As a result, registries have the potential to considerably lower dementia costs, while also improving diagnostic and care standards [5].

### Socio demographic and clinical characteristics Education

One of the most significant results is the gap in educational attainment between men and women as well as between Qatari and non-Qatari patients. Females and Qataris were more likely to have no formal education, which might be attributed to past gender and cultural norms impacting educational access, particularly among the elderly. Low educational attainment has been linked to an increased risk of dementia and other cognitive problems, most likely due to decreased cognitive reserve, which renders the brain more susceptible to pathological changes associated with aging [17,18]. These findings show that educational interventions aimed at at-risk individuals may play an important role in reducing the prevalence of cognitive impairment.

### Marital status

Our study showed a preponderance of marital status among men compared to women. Evidence from the literature shows that marital status has an impact on cognitive health because married people often benefit from increased social support, which acts as a protective factor against cognitive decline [19, 20]. This can lead to an increased prevalence of MCI and Dementia among this group.

### Vascular risk factors

In this study, MCI was predominant in both sexes, aligning with worldwide patterns indicating that MCI is a common condition among older adults [21]. However, the slightly higher incidence of dementia observed in males could be attributed to their increased exposure to vascular risk factors, including hypertension and tobacco use, which are more prevalent in male patients [22]. These findings highlight the need to control vascular risk factors in men to delay the progression of MCI to dementia.

### Ethnicity

Non-Qatari residents had higher educational attainment. The majority were clustered in urban areas, particularly in Doha. This enhances access to memory clinics, dementia screening, and treatment [23]. The higher rates of mental health issues could be attributed to the challenges associated with expatriate life, including social isolation and financial stressors, in this group [24].

### Metabolic risk factors

Qataris had a higher rate of diabetes and dyslipidaemia, both of which contribute significantly to cognitive decline [25]. This highlights the necessity for focused initiatives that address these conditions, which will benefit Qatari people. The principal cause may be a lack of physical exercise and poor eating habits [26]. There was a sex difference in the risk variables; obesity was more frequent in women. Obesity is a risk factor for cognitive decline and dementia, which can be managed by lifestyle changes [27]. Smoking is a risk factor for dementia, and is more common in men than in women. This emphasises the importance of personalised smoking cessation programs [28].

### Polypharmacy

Around half of the study population had polypharmacy, it was higher among males than females. Previous studies have reported that polypharmacy is associated with an increased likelihood of adverse drug reactions, which may contribute to cognitive decline, particularly in older adult populations [29].

### Dementia Risk Reduction

The current study highlights the need to control modifiable risk variables to prevent or delay dementia, as outlined in the World Health Organization (WHO) guidelines for dementia risk reduction [30]. The World Health Organization promotes a holistic strategy that includes increased physical activity, a balanced diet, control of cardiovascular risk factors (such as hypertension and diabetes), abstinence from tobacco, and excessive alcohol use. Furthermore, the WHO emphasises the need of social involvement and cognitive stimulation in improving cognitive health [30].

Given the growing prevalence of modifiable risk factors, including obesity, diabetes, and physical inactivity, FINGER trial multidomain interventions customized to the local environment might be especially effective [31]. Expanding the FINGER trial concepts worldwide, as represented by the World-Wide FINGERS (WW-FINGERS) effort, may produce significant insights and practical dementia prevention methods for a variety of populations, including those in Qatar and the larger Middle Eastern areas [31, 32].

### Strengths and limitations of this study

The present study focuses on the creation of a prospective memory clinic registry in Qatar, which marks a significant step in dementia research. This registry collects a complete set of demographic, clinical, and epidemiological data, thereby filling a significant gap in the existing body of research. The current study's data are baseline data, making it difficult to demonstrate causal correlations between risk variables and dementia outcomes.

## Conclusion

The findings of this study highlight the differences in dementia risk factors between genders and races, highlighting the need for customized interventions. Furthermore, the registry is a great resource for policymakers and healthcare professionals, providing evidence-based suggestions to improve dementia care, increase the well-being of patients and caregivers, and maximize resource allocation.

### Future scope of the study

This prospective registry study was conducted in memory clinics. Therefore, more long-term follow-up studies and clinical trials are required.

### What is already known on this topic?

There is limited research in the Middle Eastern area, emphasizing the importance of regional dementia registries.

### What this study adds:

This study is the first complete analysis of a prospective memory clinics registry in Qatar, providing critical information about the demographic and clinical features of memory clinics patients in the region.

## Figures and Tables

**Table 1:** Gender wise comparison memory clinics registry variables

Gender	Male (n=252)	Female (n=212)	Total (n=464)
**Qatari**	95 (37.7)	109 (51.4)	204(44)
**Non-Qatari**	157 (62.3)	103 (48.6)	260(56)
**Level of Education**			
**No formal education**	30 (11.9)	65 (30.7)	95 (20.5)
**Primary**	31 (12.3)	30 (14.2)	61 (13.1)
**Secondary**	49 (19.4)	42 (19.8)	91 (19.6)
**Tertiary**	70 (27.8)	36(17)	106 (22.8)
**Post -graduation**	29 (11.5)	8 (3.8)	37(8)
**Marital Status**			
**Divorced**	1 (0.4)	3 (1.4)	4 (0.9)
**Married**	230 (91.3)	138 (65.1)	368 (79.3)
**Single**	1 (0.4)	6 (2.8)	7 (1.5)
**Widowed**	7 (2.8)	39 (18.4)	46 (9.9)
**Geographical Zone**			
**Inside Doha**	119 (47.2)	102 (48.1)	221 (47.6)
**Outside Doha**	129 (51.2)	104 (49.1)	233 (50.2)
**Diagnosis**			
**Amnestic disorder**	2 (0.8)	0 (0)	2 (0.4)
**Delirium/post delirium**	0 (0)	4 (1.9)	4 (0.9)
**Dementia**	50 (19.8)	40 (18.9)	90 (19.4)
**Mild neurocognitive disorders**	155 (61.5)	135 (63.7)	290 (62.5)
**Other diagnosis**	31 (12.3)	25 (11.8)	56 (12.1)
**Under evaluation**	14 (5.6)	8 (3.8)	22 (4.7)
**Family History of Dementia**	25 (9.9)	30 (14.2)	55 (11.9)
**Any psychiatric comorbidity**	25 (9.9)	18 (8.5)	43 (9.3)
**Anti-depressant**	39 (15.5)	44 (20.8)	83 (17.9)
**Anti-psychotic**	12 (4.8)	9 (4.2)	21 (4.5)
**Polypharmacy**	134 (53.2)	94 (44.3)	228 (49.1)
**Carer Support**			
**Support from Family and Friends**	240 (95.2)	205 (96.7)	445 (95.9)
**Support from Private Nurse**	9 (3.6)	4 (1.9)	13 (2.8)
**BPSD**	27 (10.7)	27 (12.7)	54 (11.6)
**Risk Factors**			
**Diabetes**	154 (61.1)	110 (51.9)	264 (56.9)
**Obesity**	42 (16.7)	78 (36.8)	120 (25.9)
**Depression**	26 (10.3)	16 (7.5)	42 (9.1)
**Alcohol**	5(2)	0 (0)	5 (1.1)
**Hypertension**	175 (69.4)	133 (62.7)	308 (66.4)
**Physical Inactivity**	41 (16.3)	47 (22.2)	88(19)
**Smoking**	43 (17.1)	8 (3.8)	51(11)
**Dyslipidaemia**	126(50)	99 (46.7)	225 (48.5)
**Hearing loss**	32 (12.7)	29 (13.7)	61 (13.1)
**Traumatic Brain Injury**	2 (0.8)	6 (2.8)	8 (1.7)
**Social Isolation**	3 (1.2)	1 (0.5)	4 (0.9)
**Delirium**	4 (1.6)	9 (4.2)	13 (2.8)
**Mortality**	1 (0.4)	0 (0)	1 (0.2)

**Table 2:** Ethnicity wise comparison memory clinics registry variables

Gender	Qatari (n=204)	Non-Qatari (n=260)	Total n (%)
**Male**	95 (46.6)	157 (60.4)	252 (54.3)
**Female**	109 (53.4)	103 (39.6)	212 (45.7)
**Level of Education**			
**No formal education**	65 (31.9)	30 (11.5)	95 (20.5)
**Primary**	32 (15.7)	29 (11.2)	61 (13.1)
**Secondary**	35 (17.2)	56 (21.5)	91 (19.6)
**Tertiary**	29 (14.2)	77 (29.6)	106 (22.8)
**Post -graduation**	9 (4.4)	28 (10.8)	37(8)
**Geographical Zone**			
**Inside Doha**	82 (40.2)	139 (53.5)	221 (47.6)
**Outside Doha**	120 (58.8)	113 (43.5)	233 (50.2)
**Marital Status**			
**Divorced**	1 (0.5)	3 (1.2)	4 (0.9)
**Married**	164 (80.4)	204 (78.5)	368 (79.3)
**Single**	2(1)	5 (1.9)	7 (1.5)
**Widowed**	26 (12.7)	20 (7.7)	46 (9.9)
**Diagnosis**			
**Amnestic disorder**	1 (0.5)	1 (0.4)	2 (0.4)
**Delirium/post delirium**	2 (1.0)	2 (0.8)	4 (0.9)
**Dementia**	48 (23.5)	42 (16.2)	90 (19.4)
**Mild neurocognitive disorders**	129 (63.2)	161 (61.9)	290 (62.5)
**Other diagnosis**	16 (7.8)	40 (15.4)	56 (12.1)
**Under evaluation**	8 (3.9)	14 (5.4)	22 (4.7)
**Family History of Dementia**	20 (9.8)	35 (13.5)	55 (11.9)
**Any psychiatric comorbidity**	14 (6.9)	29 (11.2)	43 (9.3)
**Anti-depressant**	30 (14.7)	53 (20.4)	83 (17.9)
**Anti-psychotic**	9 (4.4)	12 (4.2)	21 (4.5)
**Polypharmacy**	111 (54.4)	117(45)	228 (49.1)
**Carer Support**			
**Support from Family and Friends**	191 (93.6)	254 (97.7)	445 (95.9)
**Support from Private Nurse**	10 (4.9)	3 (1.2)	13 (2.8)
**BPSD**	24 (11.8)	30 (11.5)	54 (11.6)
**Risk Factors**			
**Diabetes**	133 (65.2)	131 (50.4)	264 (56.9)
**Obesity**	52 (25.5)	68 (26.2)	120 (25.9)
**Depression**	11 (5.4)	31 (11.9)	42 (9.1)
**Alcohol**	1 (0.5)	4 (1.5)	5 (1.1)
**Hypertension**	144 (70.6)	164 (63.1)	308 (66.4)
**Physical Inactivity**	36 (17.6)	52(20)	88(19)
**Smoking**	14 (6.9)	37 (14.2)	51(11)
**Dyslipidaemia**	113 (55.4)	112 (43.1)	225 (48.5)
**Hearing loss**	28 (13.7)	33 (12.7)	61 (13.1)
**Traumatic Brain Injury**	6 (2.9)	2 (0.8)	8 (1.7)
**Social Isolation**	1 (0.5)	3 (1.2)	4 (0.9)
**Delirium**	9 (4.4)	4 (1.5)	13 (2.8)
**Mortality**	1 (0.5)	0 (0)	1 (0.2)
